# Development and initial validation of a clinical measure to assess symptoms of post-stroke depression in stroke patients at the rehabilitation stage

**DOI:** 10.3389/fpsyg.2022.928257

**Published:** 2022-07-28

**Authors:** Junya Chen, Jing Liu, Yawei Zeng, Ruonan Li, Yucui Wang, Weiwei Ding, Junyi Guo, Haiyun Lin, Jufang Li

**Affiliations:** ^1^School of Nursing, Wenzhou Medical University, Wenzhou, China; ^2^Division of Emergency Medicine, The First Affiliated Hospital of Wenzhou Medical University, Wenzhou, China; ^3^Department of Nephrology, The First Affiliated Hospital of Wenzhou Medical University, Wenzhou, China; ^4^Department of Rehabilitation, Second Affiliated Hospital and Yuying Children’s Hospital of Wenzhou Medical University, Wenzhou, China; ^5^Department of Rehabilitation, The First Affiliated Hospital of Wenzhou Medical University, Wenzhou, China

**Keywords:** post-stroke depression, content validity, instrument development, Delphi panel, rehabilitation stage

## Abstract

**Background:**

The high incidence of post-stroke depression (PSD) during rehabilitation exerts a negative effect on the treatment and functional recovery of patients with stroke and increases the risk of mortality. It is necessary to screen PSD in the rehabilitation stage and thus provide effective intervention strategies. However, existing measurements used to assess PSD in the rehabilitation stage in patients with stroke lack specificity. This study aimed to develop a clinical measure to assess symptoms of PSD in the rehabilitation stage.

**Methods:**

The research team created the initial items through a literature review and semi-structured interviews of patients with stroke. Then, the symptom-related items were estimated by three panels: healthcare professionals (*N* = 41), Delphi experts (*N* = 15), and patients with stroke in the rehabilitation stage (*N* = 30).

**Results:**

The literature review and semi-structured interview produced 51 symptom-related items including six domains, and the items were reduced to 47 by the healthcare professionals. The symptom-related items were further reduced to 33 items by a two-round Delphi consultation. The initiative coefficients of the two Delphi rounds were 71.4 and 100%, the expert authority coefficients were both 0.85, Kendall’s *W* were 0.152 and 0.408 (*p* < 0.01), and the coefficient of variation (CV) were 0.05–0.32 and 0.00–0.18, respectively. The item-level content validity index (I-CVI) was 0.53–1.00, the scale-level CVI/universal agreement (S-CVI/UA) was 0.26, and the S-CVI/average (S -CVI/Ave) was 0.85 for the first found Delphi consultation; the I-CVI was 0.67–1.00, the S-CVI/UA was 0.61, and the S-CVI/Ave was 0.97 for the second round Delphi consultation. All content validity indicators have been significantly improved compared with the first round. Using mean ≥ 4 and full score ≥ 0.5, combined with CV ≤ 0.16 as the item criteria, a clinical measure of PSD with 33 items and 6 dimensions (cognition, sleep, behavior, emotion, body, and guilt) was finally formed after two rounds. The patients with stroke made no further revisions after evaluation.

**Conclusion:**

The research team developed a specific tool with good content validity to assess the symptoms of PSD in the rehabilitation stage.

## Introduction

Post-stroke depression (PSD) is a serious and common complication after stroke, which mostly presents with a depressed mood, anhedonia, and lack of sleep and exerts negative effects on patients with stroke (Hirt et al., 2020; Wang et al., 2020). Approximately, one-third of stroke survivors suffer from PSD at different stages after stroke, which include the early stage, the rehabilitation stage, and the sequelae stage (Towfighi et al., 2017). However, other studies have shown that the incidence of PSD during the rehabilitation stage is much higher than in other periods after stroke (Dafer et al., 2008; Haq et al., 2010). Rehabilitation stage PSD refers to the PSD that occurred in patients with stroke during their rehabilitation stage (stroke within 1–6 months) (Huang and Guo, 2001). Rehabilitation stage PSD not only plays a negative effect on patient rehabilitation but also reduces patients’ level of functional independence (Paolucci et al., 1999; Ezema et al., 2019), and stroke survivors have an increased risk of mortality where rehabilitation stage PSD occurs (Cai et al., 2019). Thus, it is particularly important to assess the rehabilitation stage of PSD given the increased risk of mortality and the poor prognosis associated with PSD (Yang et al., 2017).

Presently, three types of screening tools, namely the Diagnostic and Statistical Manual of Mental Disorders-IV (DSM-IV), the ordinary depression rating scales, and the specific rating scales for PSD, are used to assess the rehabilitation stage PSD. However, they all have certain shortcomings when screening the rehabilitation stage PSD. For DSM-IV, it may be misdiagnosed when used to screen for PSD in the recovery stage, and some non-PSD patients will be diagnosed as PSD (Yue et al., 2015). It is over-professional and can only be used by professional physicians, it is not suitable to be used by untrained nurses (Cinamon et al., 2011). The ordinary depression rating scales, such as the Hamilton Depression Rating Scale (HAMD) and the Beck Depression Inventory II (BDI-II), are not specifically designed for patients with stroke and may lack specificity and sensitivity. The items in these scales may be unable to assess all the specific symptoms of rehabilitation stage PSD (Almeida and Almeida, 1999; Wang et al., 2011). In terms of the specific rating scales for PSD, such as the Post-Stroke Depression Rating Scale (PSDRS), the Yale–Brown Single-Item Screening Question, and the Post-Stroke Depression Scale (PSDS), the disadvantages are as follows. The evaluation results of PSDRS are greatly affected by age, and there is no demarcation point, which is not conducive to the judgment of PSD (Quaranta et al., 2012; Przewoznik et al., 2016). The Yale–Brown Single-Item Screening Question has only one item: “are you depressed?,” which cannot fully grasp the symptoms of the rehabilitation stage PSD and thus is unable to guide subsequent management (Watkins et al., 2007). The sample size during the development of the PSDS was small, and its reliability and validity need to be further verified when used in the assessment of the rehabilitation stage PSD (Yue et al., 2015).

**FIGURE 1:**
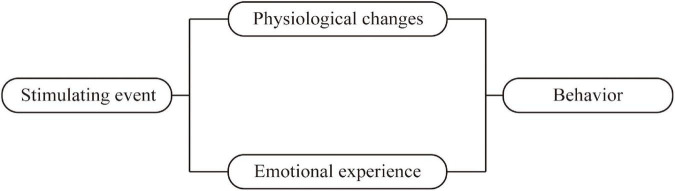
Cannon–Bard theory of emotion.

**FIGURE 2:**
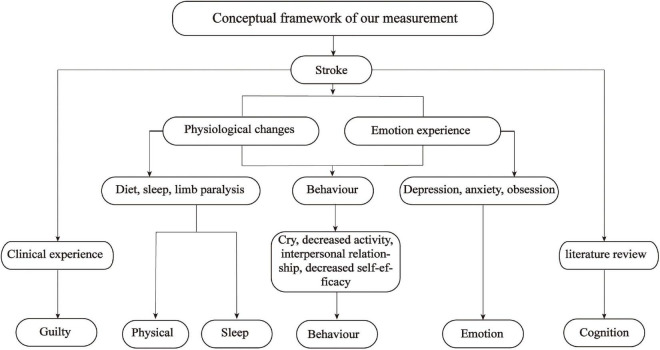
Conceptual framework for the measurement development.

In summary, the shortcomings described above limit their use to assess the rehabilitation stage PSD. The pathogenesis of PSD is different at different stages and the symptoms of PSD also vary by period (Wei et al., 2015), which generates an urgent need for the development of a specific screening tool for rehabilitation stage PSD. The Cannon–Bard theory of emotion was employed as the theoretical basis for this study (Cannon, 1929). According to the Cannon–Bard theory, emotion is produced by the thalamus after the body encounters stimulation (events), and it is related to physical changes. Physical changes and emotion are two independent components that bring different changes to individual activities as shown in Figure 1. Stroke as a stimulus event will bring corresponding physical changes and emotional experiences to patients, which in turn causes changes in patient behavior. According to the Cannon–Bard theory of emotion, the conceptual framework of this study is constructed as shown in Figure 2. The conceptual framework shows that the items of the PSD screening scale were mainly constructed from three aspects: physical change, emotional experience, and behavior. Specifically, the physical changes of patients with stroke are diet, sleep disorders, limb paralysis, and so on, which were concluded to be two subaspects of physical change and sleep change; the emotional experience includes depression, anxiety, and obsession. The physical and behavioral changes lead to a series of behavioral changes, such as crying, decreased activity, and interpersonal relationship. In addition, the literature review found that there was cognitive impairment in patients with PSD during rehabilitation, thus cognition is added as an independent aspect to the conceptual framework. Furthermore, we conclude from the relevant clinical practice experience of the research team members that guilty is common in patients with stroke, thus it is also added to the conceptual framework as one independent aspect (Figure 2). To sum up, this study aimed to develop a specific screening tool for patients with stroke in the rehabilitation stage from the aspects of cognition, physical change, sleep change, behavior, emotion, and guilty; and further evaluate its content validity by panels of healthcare professionals, Delphi experts, and patients with stroke in the rehabilitation stage.

## Materials and methods

### Study phase

Through a period of 5 months from February to June 2020, we used four steps to develop and validate the screening tool. First, we developed an initial pool through a literature review, a semi-structured interview of patients with stroke, and the existing screening tools. Then 43 healthcare professionals were invited to review the initial item pool and evaluate the relevance of each symptom-related item independently. All the symptom-related items that were rated as relevant by the healthcare professionals were then rated by a two-round Delphi expert panel including 15 experts. After the Delphi rounds, the items were evaluated by a panel of patients with stroke in the rehabilitation stage. Except for evaluating the relevance of existing symptom-related items, all the evaluators also made comments on each symptom-related item, suggested linguistic expression, reduced the items, or suggested adding new symptoms. The first phase of the study was qualitative, the second and fourth phases of the study were cross-sectional studies, and the third phase of the study was the Delphi method. Except for the Delphi consultation, other phases of this study were set in the hospital.

#### Instrument development phase 1: Establishment of the initial item pool

The initial item pool was constructed by a literature review, a semi-structured interview of patients with stroke in the rehabilitation stage, and from the items in the commonly used depression rating scales (Hamilton, 1967; Montgomery and Asberg, 1979; Zigmond and Snaith, 1983; Sheikh and Yesavage, 1986; Maboney et al., 1994; Gainotti et al., 1997; Richter et al., 1998; Quaranta et al., 2008; Li et al., 2016). In the literature review, we searched the databases Web of Science, PubMed, CNKI, Wanfang Database, and VIP database, with literature search formulas such as (“Stroke” OR “Apoplexy” OR “Cerebral infarction” OR “Cerebral hemorrhage” OR “Cerebral ischemia” OR “Cerebrovascular”) and (“Depression” OR “Depressive Disorder”) and (“Evaluation” OR “Measurement” OR “scale”). Relevant articles were imported into Endnote X9 to remove duplicates. Then, a preliminary screening is carried out according to the title and abstract of the article. Finally, the significantly relevant articles were read carefully, and relevant items were summarized from the articles. In addition, the research team conducted semi-structured interviews with 10 patients with stroke in the rehabilitation stage who had a Patient Health Questionnaire-9 (PHQ-9) score greater than or equal to 5. The purpose of the semi-structured interviews is to explore the typical symptoms of patients to supplement the item pool. The interview outline was compiled based on a literature review and research team discussion and were as follows: (i) are you adapting to the environment of the rehabilitation department of the hospital, (ii) do you have any thoughts on the rehabilitation plan formulated by the doctor, (iii) talk about the impact of stroke on your life, (iv) what you think of people around you, (v) what is your family’s attitude toward you, (vi) talk about the changes in mood during the recovery period, and did you feel down during the recovery process, when and why, (vii) during the recovery period, how did you sleep, (viii) during the hospitalization, did you feel nervous and anxious, (ix) during the recovery period, has your perspective of thinking changed, and (x) compared with your expected results, are you satisfied with the current rehabilitation treatment. Furthermore, the following commonly used depression screening scales were referred to for relevant items: the Hospital Anxiety and Depression Scale, HAMD, BDI-II, MADRS, GDS, PSDRS, Yale–Brown single-item screening question, PSDS, and Early Symptom Measurement of Post-Stroke Depression.

#### Instrument development phase 2: Healthcare professional panel evaluation

A healthcare professional panel consisting of 43 healthcare professionals rated the initial pool with 51 items. Healthcare professionals with a primary professional title or above, who has worked in the neurological department for 3 years or more, who are familiar with the treatment and nursing of patients with stroke in their rehabilitation stage, and who volunteered to participate were included in the study. The healthcare professionals rated on how common (1 = Not familiar with this item, 2 = Not seen, 3 = Rare, 4 = Common, 5 = Very common) the symptoms were observed in patients with stroke in the rehabilitation stage independently. Items rated as very common and common by more than 50% of the healthcare professionals were retained in this phase and were then evaluated by the Delphi expert (Li et al., 2018).

#### Instrument development phase 3: Delphi expert panel evaluation

The research team recruited a panel of 15 experts by purposive sampling methods (Friedman, 2010; Palinkas et al., 2015; Samuel and Kanimozhi, 2021). Experts with a bachelor’s degree or above, who worked for more than 3 years in related fields (nursing, psychology, and rehabilitation), who had an intermediate title or higher, and who volunteered to participate were included in the study.

The 47-item pool was rated by the experts who met the inclusion criteria *via* e-mail or in person, and responses were requested within 1 week. The consultation letter for experts includes instructions for filling in the form, general expert information, a consultation questionnaire, and a judgment basis. The experts rated how important (1 = Very unimportant, 2 = unimportant, 3 = Generally, 4 = Important, 5 = Very important) the symptoms were observed in patients with stroke in the rehabilitation stage independently. The Delphi results were analyzed immediately after the first round to determine if the next round of Delphi was needed. The first round of Delphi results was given back to the Delphi experts if a next Delphi consultation was needed. The second round of experts is from the experts who responded to the first round.

The indicators of Delphi are as follows: initiative coefficient, authority coefficient, coordination coefficient, the concentration of expert opinions (mean, full score ratio, and coefficient of variation), and content validity. The initiative coefficient was reflected by the response rate of the experts. The response rates were suggested over 70% or higher (Hasson et al., 2000). The degree of experts’ authority was estimated by the authority coefficient (Cr). The authority coefficient (Cr) was determined by experts’ self-rated judgment (Ca) and experts’ familiarity with the topic (Cs). Ca was calculated based on the level of theoretically sound knowledge, practical experience, collaborative or peer-based learning, and intuitive feeling (Table 1). Experts’ familiarity with the topic was rated by the experts as to how familiar they were with the symptoms (1.0 = very familiar, 0.8 = familiar, 0.5 = generally familiar, 0.2 = unfamiliar, 0 = very unfamiliar). A higher Cr indicated a high degree of trustworthiness of the Delphi experts. It is believed that Cr ≥ 0.70 is the acceptable reliability (Li et al., 2018). The coordination coefficient refers to the level of the overall agreement or consensus across the expert panel members, it was confirmed by Kendall’s coefficient of concordance (Kendall’s *W*). Kendall’s coefficient between 0.4 and 0.5 indicates a good level of consensus (Xiang et al., 2018). The concentration of the experts’ opinions includes the mean, the full score ratio, and the coefficient of variation (CV), which were used to confirm the concentration of the experts’ opinions and also were employed as the criteria for item retention. The mean is the average of the scores rated by experts on each item. The full score ratio refers to the percentage of experts who rated the items as very important to the total number of experts. The mean is higher when more experts rated the items as very important or important. The higher mean score indicates that the experts have a higher acceptable level of the item. The full score ratio ranged from 0 (no experts evaluated the item as very important) to 1 (all experts evaluated the item as very important). A higher full score ratio indicates a higher agreement of experts on the items they rate. A lower CV indicates good consensus for each item (Hou et al., 2019). Content validity indicated the level of agreement among experts. It contains the item-level content validity index (I-CVI) and the scale-level content validity index (S-CVI). I-CVI refers to the ratio of the number of experts rated the items as 4 or 5 to the total number of experts. The I-CVI should be more than 0.78 when the number of experts was more than 6 (Lawshe, 1975). The S-CVI contains the universal agreement S-CVI (S-CVI/UA) and the average S-CVI (S-CVI/Ave). The S-CVI/UA is the ratio of the number of items rated as 4 or 5 by all experts to the total number of items. S-CVI/Ave is the mean of the I-CVI. S-CVI/UA more than 0.80 and S-CVI/Ave more than 0.90 indicates excellent content validity (Lindsey, 1992).

**TABLE 1:** Development and initial validation of post-stroke depression measure for the rehabilitation stroke patients.

Phase 1: literature review	Phase 2: healthcare professional evaluation	Phase 3: delphi expert panel evaluation								Selected items
		
	Sum of very common and common (%)	First round				Second round				
			
		Mean ± SD	Full score ratio	CV	I-CVI	Mean ± SD	Full score ratio	CV	I-CVI	
**Domain and items**										
**Cognition**										
1. Memory is worse than before (such like can’t remember what for breakfast)	97.7	4.80±0.41	0.80	0.09	1.00	5.00±0.00	1.00	0.00	1.00	Selected
2. Thread is not clearer than before	93.0	4.73±0.46	0.73	0.10	1.00	4.87±0.35	0.87	0.07	1.00	Selected
3. Feel hard to concentrate	86.0	4.47±0.83	*0.73*	*0.19*	*0.80*	5.00±0.00	1.00	0.00	1.00	Selected
4. *Slow in speaking (moved to cognition domain)*	86.0	*4.13±0.83*	*0.40*	*0.20*	*0.73*	*4.00±0.76*	*0.27*	*0.18*	*0.73*	Deleted^2^
5. *Less speaking (moved to cognition domain)*	83.7	4.27±0.88	*0.60*	*0.21*	*0.73*	4.67±0.62	0.73	0.13	0.93	Selected
6. *Hard to make decisions about your own affairs (revised to “Hesitate about doing things” after Healthcare professional evaluation)*	51.2	*4.20±0.86*	*0.60*	*0.21*	*0.80*					Deleted^1^
7. Feel bored during rehabilitation	*46.5*									Deleted
8. Ruminate about one’s condition	93.0	*4.07±0.88*	*0.47*	*0.22*	*0.73*					Deleted^1^
9. Feel lose oneself (such as life, family, etc.)	79.1	4.53±0.64	0.60	0.14	0.93	4.80±0.41	0.80	0.08	1.00	Selected
10. Feel difficult to adapt transformation	83.7	*4.33±0.72*	*0.53*	*0.17*	*0.80*					Deleted^1^
11. Feel uncomfortable when people talking about my illness	65.1	*4.07±0.88*	*0.47*	*0.22*	*0.67*					Deleted^1^
*Feel gods is not fair to me about stroke*^b^**		*4.07±0.88*	*0.40*	*0.22*	*0.73*					Deleted^1^
*Need attention^a^*						*3.80±0.68*	*0.13*	*0.17*	*0.67*	Deleted^2^
**Sleep**										
12. Take longer to fall asleep	62.8	4.53±0.74	0.67	0.16	0.87	4.87±0.35	0.87	0.07	1.00	Selected
13. Get awake frequently	69.8	4.67±0.72	0.73	0.16	0.87	4.93±0.26	0.93	0.05	1.00	Selected
14. Wake up early then can’t sleep	72.1	4.73±0.70	0.80	0.15	0.87	4.87±0.35	0.87	0.07	1.00	Selected
15. Getting not enough bedtime (less than 6 hours)	58.1	*4.00±1.07*	*0.53*	*0.27*	*0.67*					Deleted^1^
16. Feel not getting enough sleep	62.8	4.33±0.82	*0.60*	*0.19*	*0.87*	4.47±0.52	0.87	0.11	1.00	Selected
17. Sleep during day and be awake at night	74.4	4.67±0.62	0.67	0.13	0.87	*4.00±0.65*	*0.20*	*0.16*	*0.80*	Deleted^2^
18. Insomnia (experts considered that repeat with 12)	79.1									Deleted
**Behavior**										
19. Unable to finish the rehabilitation plan	*46.5*	*4.27±0.88*	*0.60*	*0.21*	*0.80*					Modified
20. Get rehabilitation exercise on one own in the free time	62.8	*4.27±0.70*	*0.47*	*0.16*	*0.93*					Modified
Unable to initiate rehabilitation (modified according to the above two items after the first round)						4.20±0.77	0.40	0.18	0.80	Selected
21. Depend other’s in daily life	88.4	4.20±0.86	*0.60*	*0.21*	*0.80*	4.73*±0.59*	0.80	0.12	0.93	Selected
22. *Willpower is not firm enough when doing rehabilitation exercise (moved to behavior and revised to “Feel difficult to hang on in rehabilitation exercise” after the first round)*	76.7	3.87±0.83	*0.60*	*0.22*	*0.67*					Deleted^1^
23. Not willing to Communicate	46.5	4.60±0.74	0.67	0.16	0.80	4.93±0.26	0.93	0.05	1.00	Selected
24. Had a suicide action	*11.6*									Deleted
25. Hurt others verbally or physically	*18.6*									Deleted
26. Feel very laborious in doing everything	88.4	*4.07±0.96*	*0.47*	*0.24*	*0.73*					Deleted^1^
*Unwilling to participate in the formulation of rehabilitation plans*^a^**						4.20±0.77	0.40	0.18	0.80	Selected
**Emotion**										
27. *Feel depressed because consider oneself can’t get better (revised to “Feel depressed” by healthcare professionals)*	86.0	4.80±0.41	0.67	0.09	1.00					Combined
28. Feel down	81.4	4.93±0.26	0.67	0.05	1.00					Combined
29. Feel worried and listless	53.5									Combined
be in bad mood (combined with above three items)						4.93±0.26	0.93	0.05	1.00	Selected
30. Feel irritable	83.7	4.80±0.41	0.80	0.09	1.00	4.93±0.26	0.93	0.05	1.00	Selected
31. Can’t adjust one’s emotion	79.1	4.40±0.63	0.53	0.14	0.93	4.73±0.46	0.73	0.09	1.00	Selected
32. Being Emotional	74.4	4.40±0.63	0.53	0.14	0.93	*4.40±0.51*	*0.40*	*0.11*	*1.00*	Selected
33. *Always want to cry (revised to “want to cry or have cried” after healthcare professional evaluation)*	55.8	4.60±0.63	0.80	0.14	0.93	4.80±0.41	0.80	0.08	1.00	Selected
34. Lose interest in things surround	65.1	4.53±0.64	0.60	0.14	0.87	5.00±0.00	1.00	0.00	1.00	Selected
35. *Blame others for trifles (moved to emotional)*	48.8	*4.00±1.00*	*0.47*	*0.25*	*0.67*	4.47±0.74	0.73	0.16	0.87	Selected
36. *Think a lot when suffering insomnia (moved to emotional)*	74.4	4.67±0.62	0.80	0.13	0.87	*4.93±0.26*	*0.40*	*0.05*	*1.00*	Selected
37. Emotional changes clearly in the morning and evening	*46.5*									Deleted
*Can’t sleep because of thinking a lot*^b^* (moved to emotional and experts considered that repeat with 36)*		4.53±0.74	0.67	0.16	0.87					Deleted^1^
**Physical**										
38. *There are disparity between in comparing with the ideal state (revised to “Think that rehabilitation exercise is not working” by healthcare professionals)*	72.1	4.13±0.83	*0.80*	*0.20*	*0.80*	*4.20±0.56*	*0.27*	*0.13*	*0.93*	Deleted^2^
39. Act slow	90.7	*3.60±0.83*	*0.53*	*0.23*	*0.93*					Deleted^1^
40. *Feel fatigue more easily than before (revised to “Feel tiring to doing things” by healthcare professionals)*	90.7	4.13±0.83	*0.53*	*0.20*	*0.73*	4.80±0.41	0.80	0.08	1.00	Selected
41. Feel unpleasant (divided into the following two items after the first round)	74.4	3.67±1.18	0.80	0.32	0.53					Divided
Feel pain						*4.27±0.70*	*0.40*	*0.16*	*0.87*	Selected
Feel malaise						4.53±0.64	0.60	0.14	0.93	Selected
42. Weight changes significantly	*44.2*									Deleted
43. Eat more than before	*14.0*									Deleted
44. Don’t want to eat	*39.5*									Deleted
*Feel desperate in rehabilitation*^a^**						4.60±0.63	0.67	0.13	0.93	Selected
**Guilty**										
45. Feel drag down the family (divided into the following three items)	62.8									Divided
Feel that stroke increased financial burden on the family		4.93±0.26	0.87	0.05	0.93	*4.93±0.26*	*0.40*	*0.05*	*1.00*	Selected
Stroke diminish the quality of life		4.93±0.26	0.87	0.05	1.00	4.93±0.26	0.60	0.05	1.00	Selected
Stroke interfere with families’ work		4.80±0.56	0.80	0.12	0.93	4.93±0.26	0.67	0.05	1.00	Selected
46. *Always feel inability because can’t complete Something (revised to “Feel inability” by healthcare professionals)*	76.7	4.73±0.46	0.73	0.10	0.93	5.00±0.00	1.00	0.00	1.00	Selected
47. Blame on past bad living habits	72.1	4.20±0.94	*0.47*	*0.22*	*0.73*	4.33±0.62	0.60	0.14	0.93	Selected
48. Blame oneself for trifles	46.5	4.40±0.63	0.53	0.14	0.93	4.40±0.51	0.67	0.11	1.00	Selected
49. Consider that stroke is a punishment	*25.6*									Deleted
*Feel guilty^b^*		*4.47±0.52*	*0.47*	*0.12*	*1.00*					Deleted^1^
50. *Feel have no reason to alive (revised to “Feel that people like me deserve to die” by healthcare professionals)*	*27.9*	4.27±0.70	0.33	0.16	0.80	*4.15±0.71*	*0.60*	*0.17*	*0.80*	Selected
51. *Feel unconfident in rehabilitation(moved to guilty)*	67.4	4.53±0.64	0.53	0.14	0.93	4.90±0.29	0.67	0.07	1.00	Selected
S-CVI/UA		0.26				0.61				
S-CVI/AVE		0.85				0.97				
Initiative coefficient		0.71				1.00				
Authority coefficient		0.85				0.85				
Kendall’s W		0.152 (χ^2^ = 126.384)*				0.408 (χ^2^ = 226.682)*				
Items deleted, combined, sub-divided, new or deleted	9 deleted 1 sub-divided 3 new	10 deleted 2 combined 1sub-divided 3 new				5 deleted				
Total items retained	47 items	38 items				33 items				

Numbers in bold italics indicate unqualified index.

Wording in italics indicate item is new, moved or revised.

Mandarin to English translation preserves original Mandarin phrasing.

^a^Indicates one new item added after first Delphi round.

^b^Indicates one new item added by healthcare professionals.

^1^Indicates one item deleted after first Delphi round.

^2^Indicates one item deleted after second Delphi round.

*Indicates *P* < 0.01.

#### Instrument development phase 4: Rehabilitation stroke patient panel evaluation

In total, 30 patients with stroke in the rehabilitation stage were recruited by purposive sampling to streamline or supplement the item pool. The inclusion criteria were patients who were diagnosed with stroke and the diagnosis was further confirmed by computed tomography and magnetic resonance imaging, patients with stable vital signs and mental clarity, patients within 1–6 months of stroke, and patients who were able to communicate in written or verbal. Patients with subarachnoid hemorrhage, serious heart, liver, and renal insufficiency, cancer, unconsciousness, sensory aphasia, or cognitive impairment were excluded from this study. The stroke patient panel evaluated the 33-item pool independently in this round.

### Data analysis

IBM SPSS 26.0 was used for data analysis. Descriptive statistics including the means and standard deviations, frequencies, and percentages were used to analyze the healthcare professionals, Delphi experts’ data, and part of the items data. Specifically, the initiative coefficient was calculated as a percentage, the Cr is calculated as Cr = (Ca + Cs)/2, Kendall’s *W* was calculated by non-parametric test, and the full score ratio was calculated by percentage. CV is calculated as the ratio of mean and standard deviation. I-CVI, S-CVI/UA, and S-CVI/AVE are calculated by percentages.

### Ethical considerations

This study was approved by the ethics committee of the data collection hospital (approval number: 2020-zz-058). Informed written consent was signed by all study participants. Especially, as the scale items are related to depression, the research process may induce depression in patients with stroke. Thus, if the patient exhibited significant emotional changes, the investigation would be terminated, and a professional psychiatrist will be invited to intervene.

## Results

### Establishment of the initial item pool

The research team summarized 10 symptom-related items through the literature review and found eight new symptom-related items through the semi-structured interview of patients with stroke. Furthermore, the other 33 symptom-related items were referred from eight commonly used depression rating scales. Through the above process, an item pool with 51 symptom-related items including six domains was produced. Then the research team members conducted a discussion to determine the accuracy of the items and the 51 items were all retained for the next development step (Table 2).

**TABLE 2:** Calculated expert judgment used in authority coefficient.

Judgment based on	Impact on experts’ evaluation		
	**High**	**Moderate**	**Low**
Practical experience	0.5	0.4	0.3
Theoretical analysis	0.3	0.2	0.1
Learned from peers	0.1	0.1	0.1
Intuitive feeling	0.1	0.1	0.1
Total	1.0	0.8	0.6

### Healthcare professional evaluation

#### Demographics of the healthcare professionals

The working years of healthcare professionals ranged from 3 to 29 years (Mean = 8.47, *SD* = 6.83). In total, 26 healthcare professionals have an intermediate professional title, three got a senior title, and others got a primary title. Eight of the healthcare professionals were physical therapists, 17 were clinical neurologists, and the other 18 were registered nurses.

#### Healthcare professional evaluation of the items

In total, 40 items in the initial pool were rated as very common and common by more than 50% of the healthcare professionals, which were retained in this step. And three new items were added, and four items were modified by the healthcare professionals in this step (Table 2). Also, four items were retained after discussion by the research team although they were not rated as very common or common by the healthcare professionals. A total of 47 items were obtained in this step.

### Delphi expert panel evaluation

#### Demographics of the Delphi experts

The working years of the experts ranged from 12 to 35 years (Mean = 21.4, *SD* = 6.87). In total, 14 experts have a senior title, and one got an intermediate professional title. Three of the experts got a doctoral degree, eight of the experts got a master’s degree, and four of them got a bachelor’s degree. Five of the experts were registered nurses, four were clinical neurologists, and the other six were professors from universities. The experts were from Zhejiang, Hubei, Jiangxi, Hebei, and Shanghai provinces in China.

#### Delphi indicators

Initiative coefficient: In total, 15 of the 21 invited experts took part in the first round Delphi and then were invited to take part in the second round Delphi. None of the 15 experts dropped out of the second round Delphi. Thus, the response rates were 71.4 and 100% for the first and second rounds of Delphi consultation, respectively. Authority coefficient: The Cr in the first and second Delphi round was the same as 0.85, which indicated a high level of an expert authority. Coordination coefficient: The Kendall’s *W* in the first round Delphi was 0.152 (*p* < 0.01) and in the second round Delphi was 0.408 (*p* < 0.01) (Table 2). The Kendall’s *W* of the second round reached the level of good consensus and warranted no need for the third round Delphi consultation. The concentration of expert opinions: The mean was 3.60–4.93 in the first round and 3.80–5.00 in the second round. The full score ratio of the first round ranged from 0.33 to 0.87, and from 0.27 to 1.00 in the second round. The CV was 0.05–0.32 and 0.00–0.18, respectively. The symptom-related items with a mean less than 4.00, full score ratio less than 0.50, or CV higher than 0.16 were considered for deletion (Wei et al., 2013). Items would be deleted if they were eligible for one of the three indicators mentioned above. According to the standard, 10 items were deleted after the first round, and four items were deleted in the second round. The result is shown in Table 2. Content validity: The I-CVI of all items was above 0.78 after the second-round Delphi. The S-CVI/Ave of the first and second Delphi rounds were 0.85 and 0.97, respectively. S-CVI/UA is 0.26 after the first round and 0.61 after the second round. S-CVI/UA is affected by the number of experts, it is generally difficult to reach 0.80 if the number of experts is large (Almanasreh et al., 2019). Therefore, this indicator is allowed to be lower than 0.80.

#### Expert opinions and revisions to the items

In the first-round consultation, experts made recommendations for 24 items, and 7 experts also made comments on the whole item pool. The recommendations varied from language expression revisions (e.g., from “Willpower is not firm enough when doing rehabilitation exercise” to “Feel difficult to hang on in rehabilitation exercise”) to domain adjustment (e.g., “Slow in speaking” moved to the cognition domain). Necessary revisions were made to the items according to the suggestion by the first round of Delphi experts. After the first Delphi round, 1 item was deleted (repeat with item 36), 3 items were added, and 3 items were modified according to experts’ advice. In the second-round consultation, four experts recommended changes to nine items, and two experts commented on the whole measure. No items were modified or deleted based on expert opinions. Finally, 33 items reached a consensus after the second round of Delphi consultation (Table 2).

### Patients with stroke panel evaluation

In total, 30 patients with stroke in their rehabilitation stage were recruited according to the inclusion criteria. The age range of the participants was 37–84 years (mean = 61.53, *SD* = 12.46). Most of the participants were women (73.3%), and almost two-thirds completed 6 or more years of education. The illness duration of the participants ranged from 30 to 180 days (mean = 30.00, *SD* = 53.97), and all of them suffered from their first-ever stroke. The patients with stroke made no further revisions to the screening tool.

## Discussion

Through a literature review, a semi-structured interview, and references to existing depression rating scales, a specific screening tool with 51 items to assess the rehabilitation stage PSD was developed. Initial validation by the healthcare professional panel, the Delphi expert panel, and the patient with stroke panel resulted in a 33-item measurement with an acceptable level of consensus. And the screening tool may be the potential to effectively assess the rehabilitation stage PSD and thus provides a basis for formulating targeted nursing strategies for patients with stroke in their rehabilitation stage.

### Content validity of the screening tool

A two-round Delphi consultation reached an acceptable consensus on the screening tool for the rehabilitation stage PSD. The number of 15 experts recruited in the two-round Delphi is enough to get a stable consensus in that the increase in the number of experts has little effect on the prediction accuracy when the expert’s number is close to 15 (Wu and Sun, 2015). The rich clinical experience and professional knowledge of the Delphi experts ensured the credibility of the consulting results and the multi-regional of the Delphi experts ensured the generalizability of the consulting results. The increasing response rate from the first to the second round of Delphi consultation indicated that the experts who participated in the consultation were highly motivated and their responses were reliable (Macdonald et al., 2000). The high authority coefficient (0.85) of two rounds of Delphi consultation indicated that the experts were familiar with the symptoms and their rating on the items is reliable (Wu et al., 2019). The coordination coefficient increased significantly from the first round to the second round and reached the threshold for stopping the Delphi consultation. It indicated that the amendments proposed by experts in the first round have a high degree of consistency. Also, the coordination coefficient in the second round is higher compared to other similar studies (Zhou et al., 2019), which means that the agreement between the experts on the symptom-related items has higher consistency than other studies. The content validity indicators did increase after the two-round Delphi consultation, and all the indicators reached the standard criteria for good content validity, which indicated that all the symptom-related items were highly representative of the rehabilitation stage PSD.

### Items deleted from the development phases

A total of 21 items were deleted from the development phases. For the healthcare professional evaluation, 11 items did not meet the item retention criteria, 7 were removed, and 4 were retained based on their clinical relevance. Specifically, the item “Unable to finish the rehabilitation plan” may be slightly lower than the standard, which was revised and retained after discussion; the item “Not willing to Communicate” was obtained through a literature review (Li et al., 2018), and it was also presented by the participants in the semi-structured interview, so it was kept in the item pool; the items “Blame others for trifles” and “Blame oneself for trifles” were obtained through the semi-structured interviews, considering the importance of the stakeholders’ feelings when examining their psychological status, they were retained and to be rated by the Delphi expert panel.

For the Delphi expert panel evaluation, the mean, the full score ratio, and the CV were used as the criteria for item selection. In the first round, 19 items did not meet the item retention criteria, of which 10 were removed and 9 were retained. Specifically, the items “Feel hard to concentrate,” “Feel not getting enough sleep,”, “Blame on past bad living habits,” “Depend on others in daily life,” “Think that rehabilitation exercise is not working,” “Slow in speaking,” “Less speaking,” “Willpower is not firm enough when doing rehabilitation exercise,” and “Blame others for trifles” were retained for their clinical relevance. In addition, one item “Can’t sleep because of thinking a lot” was removed based on expert opinion that this item repeated with item 36.

In the second round, 8 items did not meet the item retention criteria, 4 were removed, and 4 were retained. The items “Being Emotional” and “Think a lot when suffering insomnia” were retained because they were observed by 74.4% of the healthcare professionals working in the neurological department. The items “Feel pain” and “Feel that stroke increased financial burden on the family” were retained because they were proposed by a psychiatrist who was very familiar with the symptoms of PSD. After the second round of Delphi consultation, the items with different opinions from experts decreased compared with the first round, which further illustrates the effectiveness and reliability of the Delphi process. Finally, no item was deleted for the stroke patient’s evaluation.

### Items added in the development phases

A total of 6 items were added in the development phases. Three items were added by the healthcare professional evaluation, which was “Feel God is not fair to me about stroke,” “Can’t sleep because of thinking a lot,” and “Feel guilty.” They were added by the clinical nurses according to their rich clinical experience and familiarity with patients with stroke. Besides, 3 items were added according to the Delphi expert advice, which was “Need attention,” “Unwilling to participate in the formulation of rehabilitation plans,” and “Feel desperate in rehabilitation.” Since the research team conducted a semi-structured interview of the patient with stroke in their rehabilitation stage during the establishment of the item pool, no relevant symptoms were added by the patient panel evaluation.

### Strengths of the final scale

Compared with the three types of screening tools currently used to evaluate rehabilitation stage PSD, the tool specially developed in this study for screening rehabilitation stage PSD has obvious advantages. First, this tool is simple to use and takes less time, so it is more suitable for routine screening of rehabilitation stage PSD than DSM-IV. Second, because the tool is specially designed for the stroke population in China, it is more sensitive and specific for PSD screening than the general depression screening scales (HAMD, BDI-II, MADRS, GDS, and others). Third, compared with the specific rating scales for PSD, this tool is not only more targeted but also can describe the symptoms of PSD more comprehensively, laying a foundation for the management of PSD during the rehabilitation stage. Finally, it is worth mentioning that we have items related to the activities of daily living because some scholars have pointed out that the patient’s activities of daily living are strongly correlated with PSD (Ghaffari et al., 2020, 2021).

## Limitations

Although this study established the good content validity of the measurement, this is only the first step in the development of this specific tool. And several limitations should be noted in this study. First, there are relatively many items on the scale at present, and a follow-up study will use statistical methods to further refine the items. Second, the measurement development steps in this study cannot prove whether this scale can effectively distinguish patients with PSD and non-PSD in the rehabilitation stage, and research regarding the discriminative validity of the scale is on the way. Third, the psychometric properties of the measurement from the statistical perspective were not tested, therefore, future research is needed to test the reliability and validity of the measurement in a statistically driven approach. Fourth, the generalizability of this measurement is limited by the cultural background, and future research is needed to test the measurement in different cultural backgrounds. Finally, a longitudinal study is also needed to test its predictive validity.

## Conclusion

This study developed a 33-item specific tool. The healthcare professionals, Delphi experts, and patients with stroke reached a consensus on the core content of PSD symptoms of patients with stroke in their rehabilitation stage. It indicated that the tool, with good content validity, would be a potential tool to assess the rehabilitation stage PSD. The finding of this study may enhance clinical assessment and raise stroke patients’ and their families’ awareness of the assessment and treatment of the rehabilitation stage PSD.

## Data availability statement

The raw data supporting the conclusions of this article will be made available by the authors, without undue reservation, to any qualified researcher.

## Ethics statement

The studies involving human participants were reviewed and approved by The First Affiliated Hospital of Wenzhou Medical University. The patients/participants provided their written informed consent to participate in this study.

## Author contributions

JC: research design and manuscript writing. RL, YZ, HL, and JG: data analysis. YW, JC, WD, and JL: data collection. JFL: research design and manuscript revising. All authors contributed to the article and approved the submitted version.
