# Corrigendum: Development and initial validation of a clinical measure to assess symptoms of post-stroke depression in stroke patients at the rehabilitation stage

**DOI:** 10.3389/fpsyg.2022.1076444

**Published:** 2022-11-04

**Authors:** Junya Chen, Jing Liu, Yawei Zeng, Ruonan Li, Yucui Wang, Weiwei Ding, Junyi Guo, Haiyun Lin, Jufang Li

**Affiliations:** ^1^School of Nursing, Wenzhou Medical University, Wenzhou, China; ^2^Division of Emergency Medicine, The First Affiliated Hospital of Wenzhou Medical University, Wenzhou, China; ^3^Department of Nephrology, The First Affiliated Hospital of Wenzhou Medical University, Wenzhou, China; ^4^Department of Rehabilitation, Second Affiliated Hospital and Yuying Children's Hospital of Wenzhou Medical University, Wenzhou, China; ^5^Department of Rehabilitation, The First Affiliated Hospital of Wenzhou Medical University, Wenzhou, China

**Keywords:** post-stroke depression, content validity, instrument development, Delphi panel, rehabilitation stage

In the published article, the **Funding** statement was missing. The correct **Funding** statement appears below.

## Funding

This study was funded by the National Natural Science Foundation of China (grant number: 71804134), the Project of Humanities and Social Sciences from the Ministry of Education in China (grant number: 18YJCZH078), and the Health Commission of Zhejiang Province (grant numbers: 2020KY631 and 2021KY780).

The authors apologize for this error and state that this does not change the scientific conclusions of the article in any way. The original article has been updated.

## Publisher's note

All claims expressed in this article are solely those of the authors and do not necessarily represent those of their affiliated organizations, or those of the publisher, the editors and the reviewers. Any product that may be evaluated in this article, or claim that may be made by its manufacturer, is not guaranteed or endorsed by the publisher.

